# Maternal vitamin D status and risk of gestational diabetes mellitus in twin pregnancies: a longitudinal twin pregnancies birth cohort study

**DOI:** 10.1186/s12937-024-00944-2

**Published:** 2024-04-10

**Authors:** Da-yan Li, Lan Wang, Li Li, Shuwei Zhou, Jiangyun Tan, Chunyan Tang, Qianqian Liao, Ting Liu, Li Wen, Hong-bo Qi

**Affiliations:** 1 Department of Obstetrics, The First Affiliated Hospital of Chongqing Medical University, Chongqing, 400016 China; 2 Department of Obstetrics and Gynecology, Banan Hospital of Chongqing Medical University, Chongqing, 401320 China; 3 Department of Obstetrics and Gynecology, Women and Children’s Hospital of Chongqing Medical University, Longshan Road 120, Yubei District, Chongqing, 401147 China; 4 Department of Obstetrics and Gynecology, Chongqing Health Center for Women and Children, Chongqing, 401147 China

**Keywords:** Vitamin D, Gestational diabetes mellitus, Twin pregnancies, China

## Abstract

**Background:**

Gestational diabetes mellitus (GDM) is a common complication of pregnancy, with significant short-term and long-term implications for both mothers and their offspring. Previous studies have indicated the potential benefits of vitamin D in reducing the risk of GDM, yet little is known about this association in twin pregnancies. This study aimed to investigate maternal vitamin D status in the second trimester and examine its association with the risk of GDM in twin pregnancies.

**Methods:**

We conducted a prospective cohort study based on data from the Chongqing Longitudinal Twin Study (LoTiS). Peripheral blood serum was collected from the mothers in the second trimester to measure 25(OH)D concentrations. GDM was diagnosed at 23–26 weeks of gestation using a 75-g 2-h oral glucose tolerance test. We used multivariable logistic regression analyses to examine the correlations between vitamin D status and the risk of GDM.

**Results:**

Of the total participants, 93 (29.9%) women were diagnosed with GDM. The mean serum 25(OH)D concentration in the second trimester was 31.1 ± 11.2 ng/mL, and the rate of vitamin D insufficiency and deficiency were 23.5% and 18.7%, respectively. Compared to women with a 25(OH)D concentration < 30 ng/mL, those with a 25(OH)D concentration ≥ 30 ng/mL had a significantly lower risk of GDM (RR 0.61; 95% CI: 0.43, 0.86), especially those who were overweight before pregnancy (RR 0.32; 95% CI: 0.16, 0.64). The restricted cubic splines model showed an inverted J-shaped relationship between vitamin D concentrations and GDM risk.

**Conclusions:**

The risk of GDM was significantly reduced in twin pregnant women with vitamin D concentrations ≥ 30 ng/mL in the second trimester.

**Trial registration:**

ChiCTR-OOC-16,008,203. Retrospectively registered on 1 April 2016.

## Background

Gestational diabetes mellitus (GDM) is defined as diabetes diagnosed in the second or third trimester of pregnancy in women who did not have clearly overt diabetes prior to gestation according to the American Diabetes Association (ADA) [1]. GDM is one of the most common pregnancy complications and exhibits varying prevalence rates ranging from 7.1% to 27.6% worldwide according to country, ethnicity and diagnostic thresholds [2]. The prevalence of GDM among the Chinese population ranges from 17.5% to 18.9% [3], while in Europe and North America, the prevalence is lower, at 7.1-7.8% [2]. GDM has been found to have short- and long-term adverse effects on both mothers and their offspring, including an increased risk of hypertensive diseases of pregnancy, cesarean deliveries and macrosomia at birth during the perinatal period, as well as a higher risk of type 2 diabetes in mothers and metabolic complications in offspring later in life [4].

Given the potential negative effects of GDM, it is crucial to identify the risk factors associated with its development. Accumulative studies have reported an association between vitamin D status and GDM prevalence [5, 6], with vitamin D deficiency being linked to an increased risk of developing GDM [7–9]. Nevertheless, it is noteworthy that all the aforementioned studies have focused primarily on singleton pregnancies, and there is a lack of comprehensive exploration of vitamin D concentrations and status in twin pregnancies and their association with the development of GDM.

With the development of assisted reproductive technology and delayed childbearing, the rate of twin births has exceeded 3% [10]. When the same diagnostic criteria are used to diagnose GDM, twin pregnancies are found to have a higher prevalence of GDM than singleton pregnancies in the same geographical region [11–13]. This may be attributed to older age, larger placental areas and greater gestational weight gain in twin pregnant women [14]. However, studies on the impact of GDM on perinatal outcomes in twin pregnancies have reported conflicting results. Studies conducted in North America revealed that GDM is associated with an increased risk of cesarean section, preterm delivery and large-for-gestational age (LGA) neonates in twin pregnancies [15–18]. However, Lin et al. reported that the perinatal outcomes of women with twin pregnancies with GDM are comparable to those without GDM in a Chinese population [19]. In our previous prospective investigation, we found that twin pregnancies with GDM are related to an elevated risk of gestational hypertension, childhood overweight at 6 months [20] and preterm delivery [21]. This suggests that GDM may affect the health of both twin pregnant women and their offspring. Therefore, it is worth exploring whether there is a correlation between vitamin D status and the occurrence of GDM in twin pregnancies.

The aim of the present study was to investigate the vitamin D concentrations and status in the second trimester and to examine their associations with the development of GDM in twin pregnancies. To achieve this goal, we utilized a longitudinal birth cohort of twin pregnancies from Southwest China.

## Methods

### Study design and participants

This study was conducted as part of the Chongqing Longitudinal Twin Study (LoTiS), which is an ongoing prospective study conducted at the First Affiliated Hospital of Chongqing Medical University and Chongqing Health Center for Women and Children in China [22]. Chongqing is located in southwestern China at a latitude of 29.35° N and has a humid subtropical monsoon climate with insufficient sunshine (1000–1400 h per year). The LoTiS study recruited twin pregnant women aged 20–40 years who began receiving prenatal care at 11–16 weeks of gestation in the study centers. The twin birth cohort was launched in January 2016; by February 2019, a total of 439 women were recruited at the first visit, and 333 women had completed all the required visits during the pregnancy period. The study was approved by the Ethics Committee of the First Affiliated Hospital of Chongqing Medical University (No. 201530). All the methods and procedures carried out in this study were in accordance with the principles of the Declaration of Helsinki as revised in 2008. Written informed consent was obtained from each participant at recruitment.

In the current study, women were eligible for inclusion if they had peripheral blood samples collected in the second trimester, underwent a 75-g oral glucose-tolerance test (OGTT) between 23 and 26 weeks of gestation, and had complete pregnancy records. Women with any of the following conditions were excluded from the study: preexisting chronic metabolic diseases, such as hypertension or type 2 diabetes; fetal complicated with severe malformation and complications, such as twin-to-twin transfusion syndrome and intrauterine death of one or both fetuses.

### Vitamin D measurement

Peripheral blood samples were collected from mothers in the second trimester (23–26 weeks of gestation) by using a coagulation-promoting blood collection tube. Serum samples were centrifuged for 10 min at 4℃ and 3000 rpm, and transferred to -80 ℃ freezers within 3 h for long-term storage. Serum 25(OH)D_3_ and 25(OH)D_2_ concentrations were measured by high-performance liquid chromatography- electrospray tandem mass spectrometry (HPLC-MS/MS, Waters, USA), which is the gold standard measurement method. The intra-assay and inter-assay coefficients of variation were < 15%, indicating good repeatability.

The concentration of 25(OH)D was calculated by summing the concentrations of 25(OH)D_3_ and 25(OH)D_2_. The women were categorized into three 25(OH)D status groups according to the Endocrine Society guidelines: 25(OH)D concentrations below 20 ng/mL were classified as deficient, concentrations ranging from 20 to 30 ng/mL were considered insufficient, and concentrations above 30 ng/mL were considered sufficient [23].

### Diagnosis of GDM

GDM was diagnosed after the 75 g 2-h OGTT if ≥ 1 of the following plasma glucose values was met or exceeded according to the International Association of Diabetes and Pregnancy Study Group (IADPSG): a fasting plasma glucose (FPG) level ≥ 5.1 mmol/L, a 1-h plasma glucose (PG-1 h) level ≥ 10.0 mmol/L, or a 2-h plasma glucose (PG-2 h) level ≥ 8.5 mmol/L [24].

### Data collection

We collected data on maternal age (< 35 y, ≥ 35 y), height, prepregnancy weight, weight at 12 weeks, weight at 24 weeks, education level (≤ 12 y, > 12 y), employment status (employed, unemployed), smoking status before pregnancy, chorionicity (monochorionic, dichorionic), mode of conception (naturally conceived, conceived by assisted reproductive technology), parity (0, ≥ 1), family history of diabetes, gestational age and season of blood sample collection (summer/autumn, winter/spring). Prepregnancy BMI (kg/m^2^) was calculated as the ratio of weight (kg) to squared height (m^2^) (< 24 and ≥ 24 kg/m^2^), which was derived from self-reported prepregnancy weight and measured height at the first visit.

### Statistical analysis

Continuous variables are expressed as the means and standard deviations and were analyzed using Student’s t test or one-way analysis of variance. Categorical variables are expressed as count and percentage and were analyzed using the chi-squared test or Fisher’s exact test. Multivariate logistic regression models were utilized to estimate the relative ratio (RR) and 95% confidence interval (CI) for GDM risk related to vitamin D status. Adjusted confounders included maternal age, prepregnancy BMI, education level, employment status, parity, mode of conception and family history of diabetes. Additionally, we employed a restricted cubic spline (RCS) regression model to further examine the nonlinear association between vitamin D concentrations and GDM risk.

All the statistical analyses were conducted in Stata 15.0 (StataCorp, College Station, TX, USA).

## Results

### Characteristics of the participants according to GDM status

**Fig. 1:**
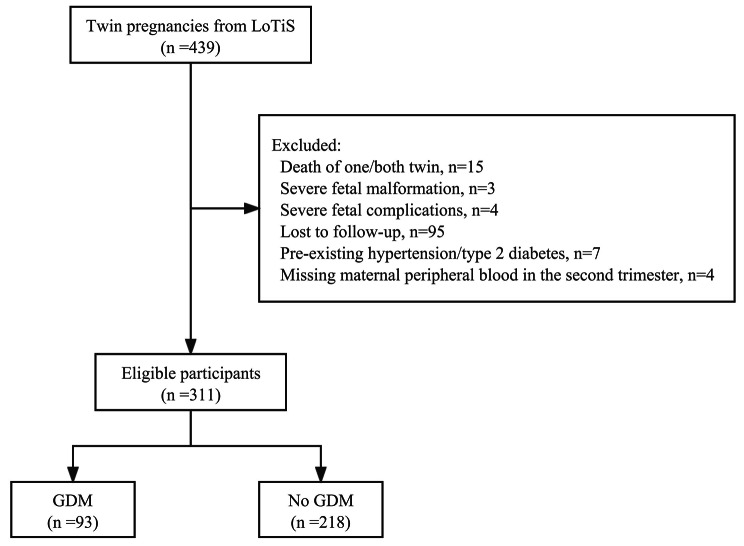
Flowchart showing selection of participants included in this analysis from LoTiS study

After exclusions of twin pregnancies due to the death of one or both twins, complicated with severe fetal malformation and complications, preexisting hypertension/type 2 diabetes, lost to follow-up and missing peripheral blood samples, a total of 311 twin pregnant women were included in the current study (Fig. 1). Among them, 93 (29.9%) were diagnosed with GDM (Fig. 1).

**Fig. 2:**
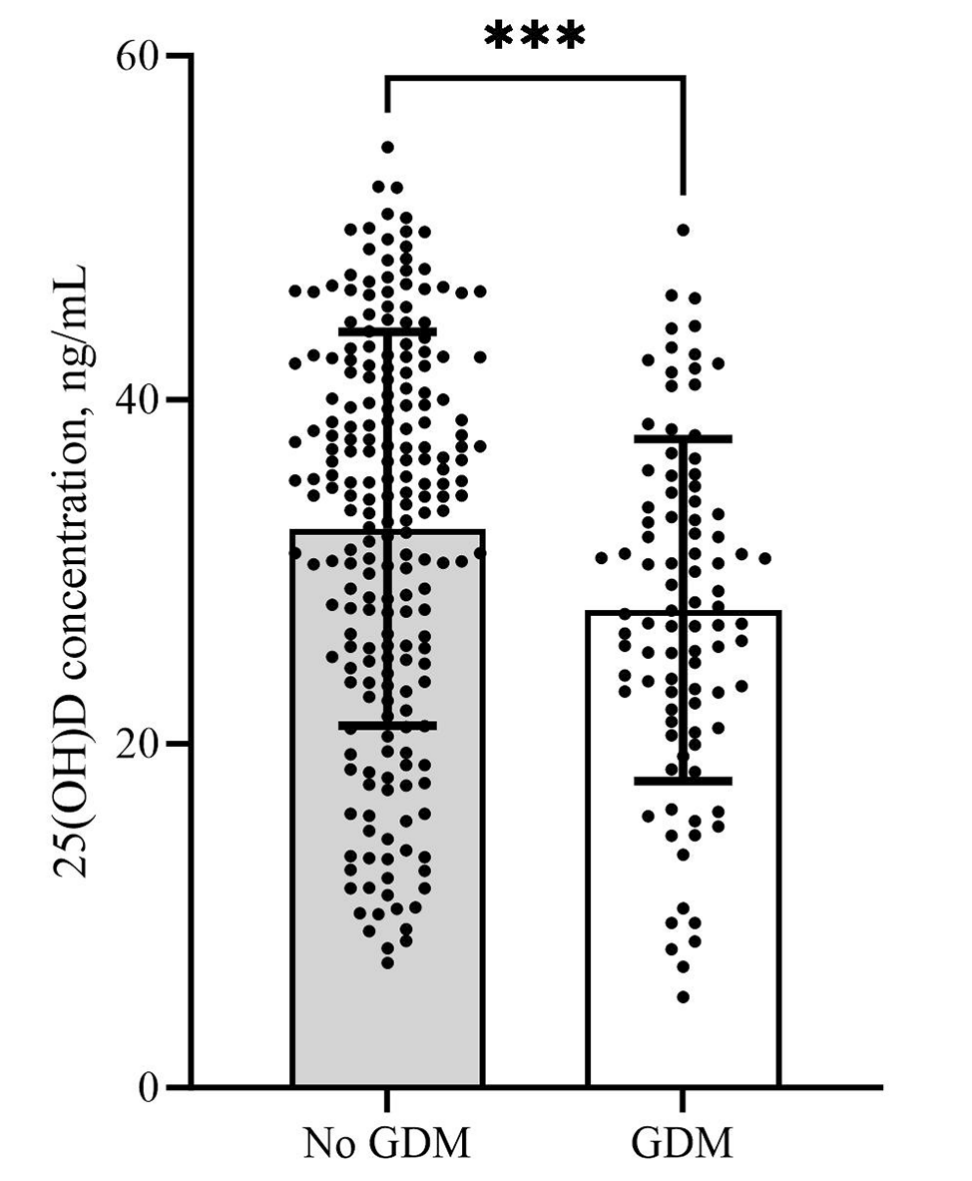
Comparison of vitamin D concentrations between women complicated with GDM and without GDM. (***) represents *p* < 0.001

Table 1 presents the participant characteristics according to GDM status. Overall, compared to women uncomplicated with GDM, women complicated with GDM tended to be older (30.0 ± 3.9 vs. 29.0 ± 3.9, *p* = 0.031) and were more likely to have a BMI higher than 24.0 kg/m^2^ before pregnancy (26.9% vs. 17.0%, *p* = 0.045). There were no significant differences between the GDM and non-GDM groups in terms of education level, employment status, primipara, mode of conception, chorionicity, smoking status before pregnancy, family history of diabetes and season of sampling. Importantly, women complicated with GDM had significantly lower concentrations of 25(OH)D and a lower proportion of vitamin D sufficiency than women uncomplicated with GDM (27.8 ± 9.9 vs. 32.5 ± 11.4, *p* < 0.001; 44.1% vs. 63.8%, *p* = 0.002). The distribution of serum 25(OH)D concentrations between the two groups is presented in Fig. 2.

**Table 1:** Clinical characteristics of the participants according to GDM status

Variables	No GDM (*n* = 218)	GDM (*n* = 93)	***P***-value
Age, years	29.0 ± 3.9	30.0 ± 3.9	0.031
Age ≥ 35 y, n (%)	16 (7.3)	17 (18.3)	0.004
Education > 12 years, n (%)	163 (74.8)	68 (73.1)	0.760
Employed, n (%)	163 (74.8)	70 (75.3)	1.000
Prepregnancy BMI, kg/m^2^	21.3 ± 2.9	22.5 ± 3.2	0.002
Prepregnancy BMI ≥ 24 kg/m^2^, n (%)	37 (17.0)	25 (26.9)	0.045
Primipara, n (%)	156 (71.6)	71 (76.3)	0.384
Conception with ART, n (%)	79 (36.2)	40 (43.0)	0.261
Monochorionic twins, n (%)	88 (40.4)	40 (43.0)	0.664
Smoking before pregnancy, n (%)	8 (3.7)	6 (6.5)	0.279
Family history of diabetes, n (%)	8 (3.7)	5 (5.4)	0.491
Gestational age of sampling, wk	24.2 ± 1.0	24.3 ± 0.8	0.124
Season of sampling			0.338
Summer/autumn, n (%)	90 (41.3)	33 (35.5)	
Winter/spring, n (%)	128 (58.7)	60 (64.5)	
25(OH)D concentrations, ng/mL	32.5 ± 11.4	27.8 ± 9.9	< 0.001
Sufficiency (≥ 30 ng/mL), n (%)	139 (63.8)	41 (44.1)	0.002
Insufficiency (20–30 ng/mL), n (%)	40 (18.4)	33 (35.5)	
Deficiency (< 20 ng/mL), n (%)	39 (17.9)	19 (20.4)	

*ART* assistant reproductive technology, *BMI* body mass index, *GDM* gestational diabetes mellitus

### Comparisons of vitamin D concentrations in the second trimester according to the maternal characteristics

As shown in Table 2, the average concentration of 25(OH)D in the second trimester was 31.1 ± 11.2 ng/mL, with vitamin D sufficiency present in 57.9% of mothers, vitamin D insufficiency in 23.5% and vitamin D deficiency in 18.7%. A significant difference in the mean 25(OH)D concentration was observed among twin pregnant women with different modes of conception. Women who conceived with the aid of assisted reproductive technology had a lower mean 25(OH)D concentration (29.3 ± 10.9 vs. 32.2 ± 11.3, *p* = 0.030). There were no significant differences in the mean 25(OH)D concentration between the other maternal characteristics and the season of sampling.

**Table 2:** The serum 25(OH)D concentration in second trimester stratifed by the characteristics of twin pregnant women

Variables	25(OH)D concentrations, ng/mL					
	Mean	*P*-value	Deficiency (< 20)	Insufficiency (20–30)	Sufficiency (≥ 30)	*P*-value
Total	31.1 ± 11.2		58 (18.7)	73 (23.5)	180 (57.9)	
Age, years		0.545				0.608
< 35	31.2 ± 11.4		52 (18.7)	63 (22.7)	163 (58.6)	
≥ 35	30.0 ± 9.8		6 (18.2)	10 (30.3)	17 (51.5)	
Education, years		0.362				0.377
≤ 12	31.4 ± 11.2		41 (17.8)	51 (22.1)	139 (60.2)	
> 12	30.1 ± 11.2		17 (21.2)	24 (30.0)	39 (48.8)	
Employment status		0.516				0.657
Employed	30.9 ± 11.1		43 (18.5)	52 (22.3)	138 (59.2)	
Unemployed	31.8 ± 11.6		15 (19.2)	21 (26.9)	42 (53.9)	
Prepregnancy BMI, kg/m^2^		0.748				0.325
< 24	31.2 ± 11.3		50 (20.1)	57 (22.9)	142 (57.0)	
≥ 24	30.7 ± 11.0		10 (16.1)	19 (30.7)	33 (53.2)	
Parity (number of deliveries)		0.077				0.505
0	30.4 ± 11.2		45 (19.8)	55 (24.2)	127 (56.0)	
≥ 1	32.9 ± 11.0		13 (15.5)	18 (21.4)	53 (63.1)	
Mode of conception		0.030				0.381
Natural conceived	32.2 ± 11.3		33 (17.2)	42 (21.9)	117 (60.9)	
ART	29.3 ± 10.9		25 (21.0)	31 (26.1)	63 (52.9)	
Chorionicity		0.105				0.515
Monochorionic	32.3 ± 11.2		22 (17.2)	27 (21.1)	79 (61.7)	
Dichorionic	30.2 ± 11.1		36 (19.7)	46 (25.1)	101 (55.2)	
Season of sampling		0.745				0.862
Summer/autumn	31.3 ± 11.1		34 (18.1)	46 (24.5)	108 (57.5)	
Winter/spring	30.8 ± 11.3		24 (19.5)	27 (22.0)	72 (58.5)	

*ART* assistant reproductive technology, *BMI* body mass index, *GDM* gestational diabetes mellitus

### Association between vitamin D status and the risk of GDM

The multivariate regression analyses performed to determine the association between vitamin D status in the second trimester and the risk of GDM are summarized in Table 3. Compared to women with vitamin D sufficiency, women with vitamin D insufficiency had a higher risk of developing GDM (RR 1.98; 95% CI: 1.37, 2.87; *p* < 0.001). After adjusting for maternal age, prepregnancy BMI, education level, employment status, parity, mode of conception and family history of diabetes, the association between vitamin D insufficiency and GDM risk remained significant (RR 1.85; 95% CI: 1.28, 2.67; *p* = 0.001). Women with vitamin D deficiency did not have an increased risk of GDM according to either the unadjusted or the adjusted model.

**Table 3:** Association between vitamin D status in the second trimester and the risk of GDM

25(OH)D status	Risk of GDM			
	Crude RR (95%CI)	*P*-value	Adjusted RR ^a^ (95%CI)	*P*-value
All participants				
Sufficiency (≥ 30 ng/mL)	1.00		1.00	
Insufficiency (20–30 ng/mL)	1.98 (1.37, 2.87)	< 0.001	1.85 (1.28, 2.67)	0.001
Deficiency (< 20 ng/mL)	1.44 (0.91, 2.27)	0.119	1.37 (0.88, 2.14)	0.168
Overweight participants				
Sufficiency (≥ 30 ng/mL)	1.00		1.00	
Insufficiency (20–30 ng/mL)	3.23 (1.55, 6.70)	0.002	3.55 (1.75, 7.20)	0.001
Deficiency (< 20 ng/mL)	2.36 (0.95, 5.86)	0.065	2.38 (1.03, 5.53)	0.043
Non-overweight participants				
Sufficiency (≥ 30 ng/mL)	1.00		1.00	
Insufficiency (20–30 ng/mL)	1.60 (1.01, 2.53)	0.043	1.47 (0.92, 2.36)	0.106
Deficiency (< 20 ng/mL)	1.26 (0.74, 2.15)	0.392	1.21 (0.72, 2.04)	0.472
Participants ≥ 35 years				
Sufficiency (≥ 30 ng/mL)	1.00		1.00	
Insufficiency (20–30 ng/mL)	3.06 (1.41, 6.65)	0.005	2.88 (1.25, 6.61)	0.013
Deficiency (< 20 ng/mL)	1.70 (0.56, 5.13)	0.346	1.67 (0.53, 5.26)	0.378
Participants < 35 years				
Sufficiency (≥ 30 ng/mL)	1.00		1.00	
Insufficiency (20–30 ng/mL)	1.72 (1.15, 2.65)	0.012	1.67 (1.09, 2.56)	0.018
Deficiency (< 20 ng/mL)	1.39 (0.84, 2.30)	0.194	1.34 (0.82, 2.18)	0.246

*GDM* gestational diabetes mellitus, *RR* relative ratio

^a^Adjusted for maternal age, prepregnancy BMI, education level, employment status, parity, mode of conception and family history of diabetes

In the subgroup analysis, a significant increase in GDM risk was observed in both the vitamin D insufficiency group (RR 3.55; 95% CI: 1.75, 7.20; *p* = 0.001) and vitamin D deficiency group (RR 2.38; 95% CI: 1.03, 5.53; *p* = 0.043) among overweight women compared to the vitamin D sufficiency group after adjustments were made for confounding factors (Table 3). Age did not modify the association between vitamin D insufficiency and GDM risk, as increased GDM risks were observed in both the vitamin D insufficiency group among both twin pregnant women aged ≥ 35 years (RR 2.88; 95% CI: 1.25, 6.61; *p* = 0.013) and those aged < 35 years (RR 1.67; 95% CI: 1.09, 2.56; *p* = 0.018) after adjusting for confounding factors (Table 3).

**Fig. 3:**
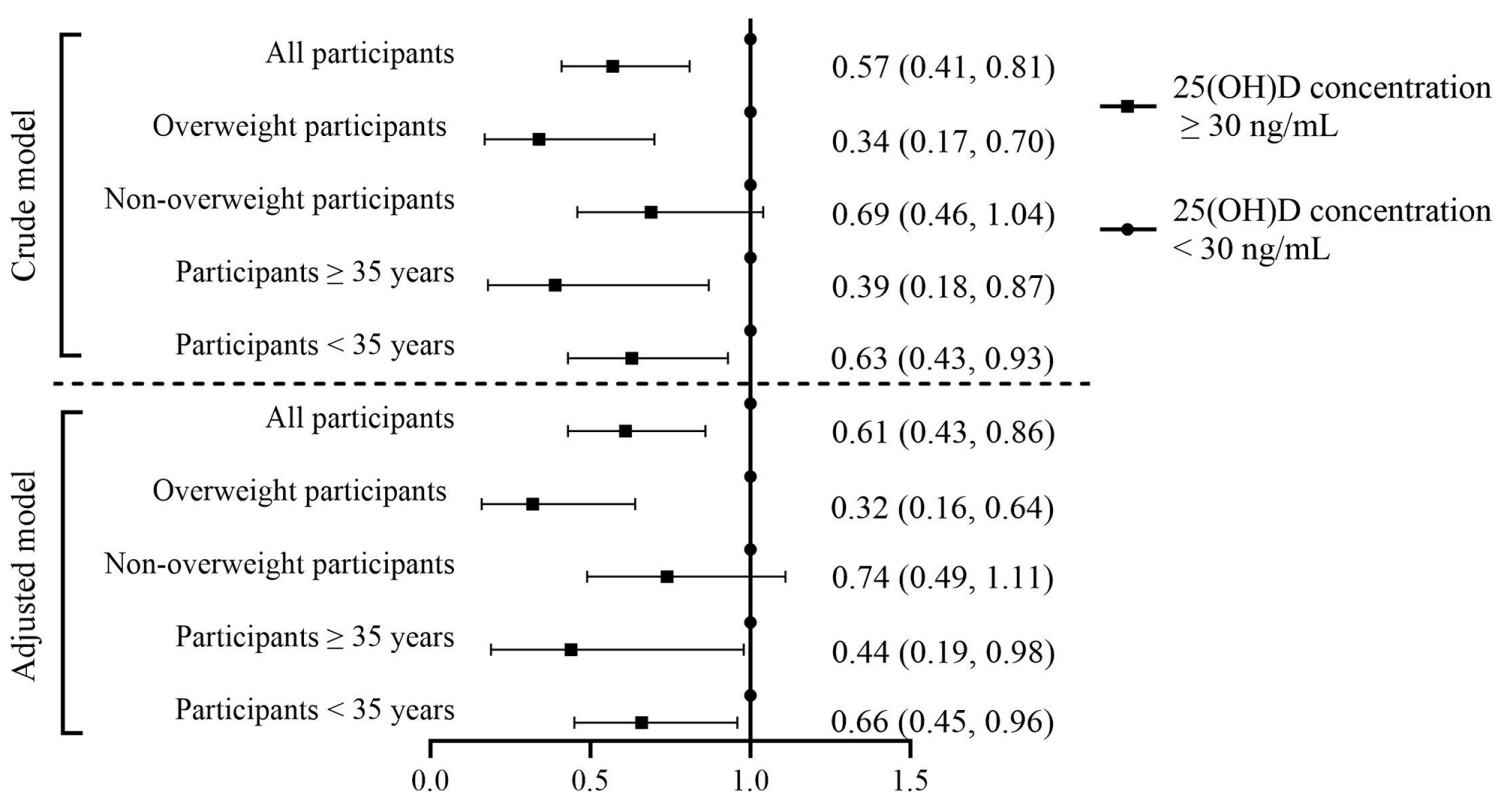
Associations between vitamin D levels and the risk of GDM, and stratified by pre-pregnancy body mass index levels (< 24.0 vs. ≥ 24.0) and age (< 35 vs. ≥ 354.0). Adjusted for maternal age, prepregnancy BMI, education level, occupation, parity, mode of conception and family history of diabetes. (●) represents vitamin D concentrations < 30 ng/mL; (■) representsvitamin D concentrations ≥ 30 ng/mL

Furthermore, we examined the association of vitamin D sufficiency with the incidence of GDM (Fig. 3). Women with vitamin D concentrations ≥ 30 ng/mL had a reduced risk of developing GDM compared to those with vitamin D concentrations < 30 ng/mL (RR 0.61; 95% CI: 0.43, 0.86; *p* = 0.005) after adjusting for potential confounding factors. The effect modification by prepregnancy BMI remained significant, as overweight women with sufficient vitamin D had a reduced risk of GDM (RR 0.32; 95% CI: 0.16, 0.64; *p* = 0.001).

### Associations between vitamin D concentrations and the risk of GDM

**Fig. 4:**
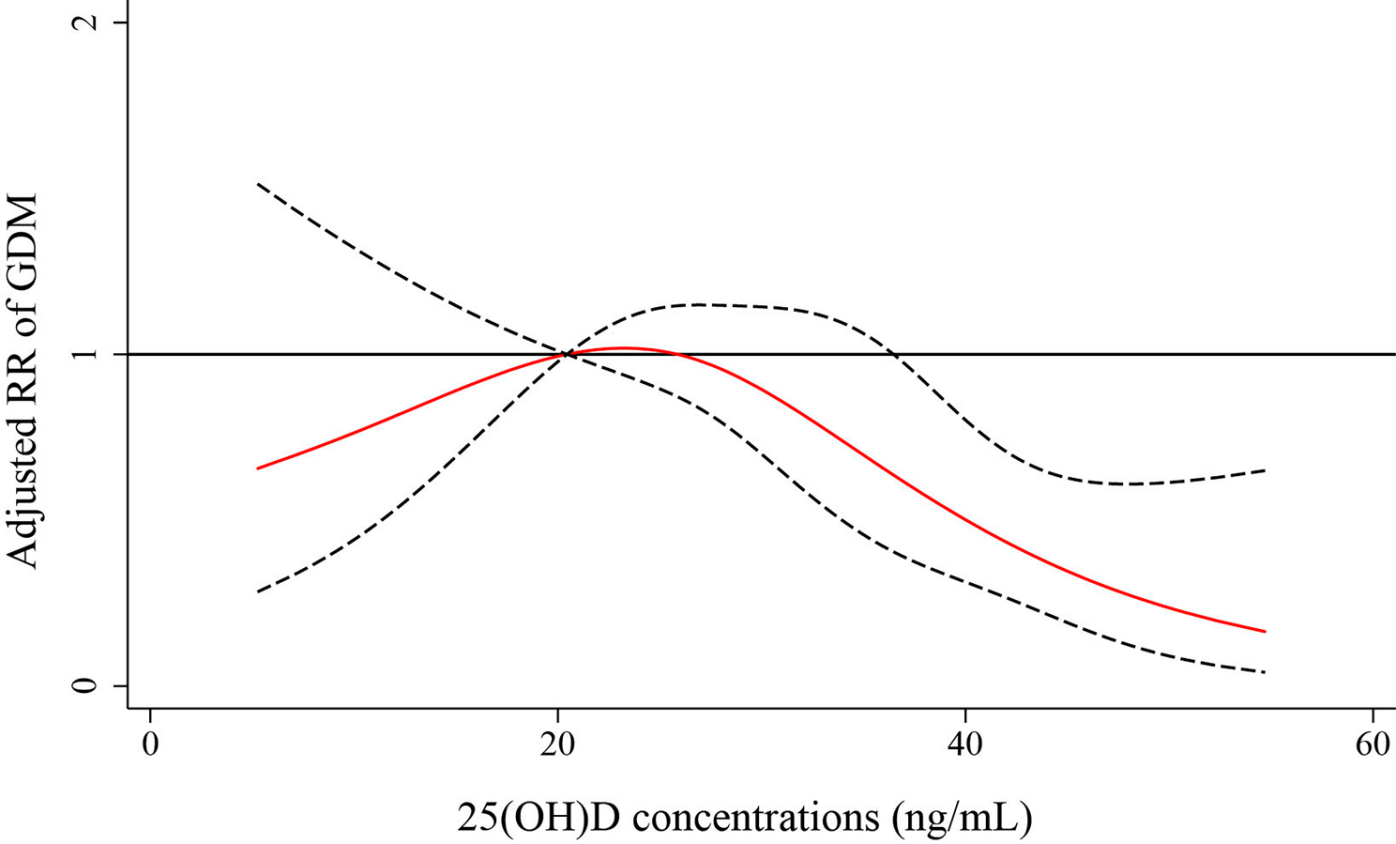
Nonlinear association between vitamin D levels in the second trimester and GDM risk by restricted cubic spline curve, maternal age, prepregnancy BMI, education level, occupation, parity, mode of conception and family history of diabetes were adjusted. A vitamin D concentration of 20 ng/mL was selected as the reference level. The area between dashed lines represents 95% CIs. Knots were located at the 5th, 35th, 65th and 95th percentiles

The RCS model showed an inverted J-shaped association between vitamin D concentrations and the risk of GDM. This association was observed after adjusting for maternal age, prepregnancy BMI, education level, employment status, parity, mode of conception and family history of diabetes (Fig. 4). Break-point analysis showed that the knot of the steep downward trend was 30 ng/mL. There was no significant association between vitamin D concentration and GDM when the 25(OH)D concentration was < 30 ng/mL, while the risk of GDM decreased when the 25(OH)D concentration was ≥ 30 ng/mL.

## Discussion

In the current cohort study, the results demonstrated that the average concentration of 25(OH)D in the second trimester among twin pregnant women was 31.1 ± 11.2 ng/mL, and 57.9%, 23.5% and 18.7% of women had sufficient, insufficient and deficient vitamin D levels, respectively. A nonlinear association between vitamin D concentrations and the incidence of GDM was observed. A vitamin D concentration above 30 ng/mL in the second trimester was found to be a protective factor against the development of GDM. This protective effect was more pronounced in twin pregnant women who were overweight prior to pregnancy.

Vitamin D deficiency is a prevalent public health issue, particularly among pregnant women. In our study, the average concentration of vitamin D in twin pregnant women in the second trimester was 31.1 ng/mL, which was higher than that observed in singleton pregnant women in China during the same trimester [25–27]. This difference may be attributed to the fact that twin pregnancies are widely recognized as high-risk pregnancies in clinical practice, leading to better compliance among twin pregnant women with prenatal health management recommendations, such as more frequent ultrasound examinations and nutritional supplementation. However, we observed that twin pregnant women who conceived with the assistance of assisted reproductive technology had lower vitamin D concentrations compare to women who conceived naturally. This may be due to the health issues commonly associated with women undergoing assisted reproductive technology, such as infertility or hormonal imbalances, traumatic procedures like embryo transfer, and the use of additional medications. These factors may impact the absorption and metabolism of vitamin D. Individual variations in vitamin D supplementation may also contribute to the observed differences.

Extensive research has been conducted on the association between vitamin D levels and the occurrence of GDM in singleton pregnancies. There are conflicting reports exist regarding the association between vitamin D levels during early pregnancy and the development of GDM. Some studies have figured out that vitamin D deficiency during early pregnancy is associated with an increased risk of GDM [7, 8, 28–30], while other studies have not supported this association [31–33]. A systematic review and meta‑analysis consisting of 37,838 pregnant women concluded that lower levels of vitamin D in early pregnancy were associated with a higher risk of developing GDM. However, in some of the included studies, vitamin D concentrations were measured in the second trimester, which limits the applicability of the findings [5]. In terms of the correlation between second trimester vitamin D levels and the occurrence of GDM, eighteen studies utilized a prospective cohort or nested case-control study design to measure vitamin D levels at 24–28 weeks of gestation. Among these studies, eleven studies reported a positive association between vitamin D deficiency and GDM risk [5, 6]. These varying results may be attributed to several factors, such as the study design, sample size, methods used to determine vitamin D levels, region and latitude. For instance, most nested case-control investigations concluded a positive association between vitamin D deficiency and higher risk of GDM. Four studies conducted on Chinese women have consistently reported an association between vitamin D levels in the second trimester and GDM risk [25–27, 34]. Hence, we assessed the vitamin D concentration in twin pregnant women in the second trimester and investigated its association with GDM.

In the current study, we discovered that vitamin D insufficiency in the second trimester was associated with an elevated risk of GDM. This association was more pronounced among overweight women, which aligns with the findings of a previous study that reported a stronger association between vitamin D and GDM risk among overweight/obese women [26]. However, we did not observe a connection between vitamin D deficiency in the second trimester and a higher risk of GDM. Therefore, we speculated that there might be a nonlinear relationship between vitamin D concentrations and GDM risk. Previous studies have shown that GDM risk was significantly reduced among pregnant women with vitamin D concentrations ≥ 20 ng/mL [25, 28], or decreased among those with vitamin D concentrations > 35 ng/mL [35], or decreased among those with vitamin D concentrations 25–40 ng/mL [32] in singleton pregnancies, suggesting the existence of a threshold concentration for vitamin D that determines the significance of its association with GDM risk. In our study, nonlinear association analysis revealed an inverted J-shaped relationship between vitamin D concentrations and the risk of GDM. A vitamin D level of 30 ng/mL was identified as the threshold that significantly reduced the risk of GDM in twin pregnant women. The variations in the identified thresholds of vitamin D, which affect GDM risk, across different studies may be ascribed to inconsistent population characteristics, diagnostic criteria for GDM and timing of vitamin D measurement.

Several biological mechanisms have been proposed to elucidate the role of vitamin D in regulating glucose metabolism. First, vitamin D may enhance the peripheral/hepatic uptake of glucose, which can help decrease glucose levels [36]. Second, vitamin D deficiency may impair pancreatic β-cell functions, thereby compromising the secretion of insulin [37]. Third, vitamin D plays a role in immune system regulation. It has been suggested that dysregulation of the immune system during pregnancy may contribute to the development of GDM, and vitamin D may help modulate immune responses and promote a balanced immune system, potentially reducing the risk of GDM. Finally, vitamin D deficiency can exacerbate inflammation and oxidative stress in the pancreas and other organs, leading to insulin resistance [37]. Compared to singleton pregnant women with GDM, twin pregnant women with GDM are more likely to have abnormal postprandial blood glucose levels, which is more likely attribute to insulin resistance than impaired pancreatic islet β cell function [21, 38–40]. Thus, higher vitamin D concentrations are significant for alleviating insulin resistance and reducing the risk of GDM in twin pregnant women. This also explains why the threshold of vitamin D, which can affect the incidence of GDM, is higher in twin pregnancies than in singleton pregnancies in the Chinese population under the same diagnostic criteria for GDM [25].

The strength of our study lies in the specific study population. To our knowledge, this was the first study to investigate the association between vitamin D status and the risk of GDM in a population of women with twin pregnancies. However, there are several limitations that should be considered in this study. One limitation was the single-center study design of this study, which limits the generalizability of the findings. Another limitation was the lack of accurate data on vitamin D supplementation during the second trimester. The questionnaire used to assess vitamin D supplementation frequency had only two options: “daily” and “sometimes or less frequently”. Further detailed investigations are needed to understand the associations among vitamin D supplementation, vitamin D absorption, and the causal mechanism underlying the relationship between vitamin D supplementation and GDM. Finally, the lack of certain biological indicators related to GDM, such as glycosylated hemoglobin and insulin, limits our ability to fully explain the effect of vitamin D on GDM.

## Conclusions

In twin pregnant women with vitamin D concentrations < 30 ng/mL in the second trimester, the risk of GDM was significantly reduced in those with vitamin D levels ≥ 30 ng/mL in the second trimester. There was a nonlinear association between vitamin D concentrations and the incidence of GDM, with 30 ng/mL considered as the cutoff for the vitamin D concentration that could significantly reduce the risk of GDM in twin pregnancies. Further multicenter research is needed to provide more evidence elucidating the relationship between vitamin D and GDM in twin pregnancies.

## Data Availability

The datasets used and/or analyzed during the current study are available from the corresponding author upon request.

## Abbreviations

ADA: America Diabetes Association
ART: assistant reproductive technology
BMI: body mass index
CI: confidence interval
GDM: gestational diabetes mellitus
IADPSG: International Association of Diabetes and Pregnancy Study Groups
LGA: large-for-gestational age
LoTiS: Longitudinal Twin Study
OGTT: oral glucose tolerance test
RCS: restricted cubic spline
RR: relative ratio
